# Meaning of self-management from the perspective of individuals with traumatic spinal cord injury, their caregivers, and acute care and rehabilitation managers: an opportunity for improved care delivery

**DOI:** 10.1186/s12883-016-0534-2

**Published:** 2016-01-23

**Authors:** Sarah E. P. Munce, Fiona Webster, Michael G. Fehlings, Sharon E. Straus, Eunice Jang, Susan B. Jaglal

**Affiliations:** Institute of Health, Policy, Management and Evaluation, University of Toronto, Rehabilitation Sciences Building, 160-500 University Ave., Toronto, M5G 1 V7 ON Canada; Department of Family and Community Medicine, University of Toronto, Toronto, Canada; Division of Neurosurgery, Department of Surgery, University of Toronto, Toronto, Ontario Canada; Li Ka Shing Knowledge Institute of St. Michael’s Hospital, Toronto, Ontario Canada; Department of Applied Psychology & Human Development, Ontario Institute for Studies in Education, Toronto, Ontario Canada; Department of Physical Therapy, University of Toronto, Toronto, Canada; Toronto Rehabilitation Institute, Toronto, Canada

**Keywords:** Meaning, Self-management, Traumatic, Spinal cord injury, Qualitative

## Abstract

**Background:**

The trend of decreasing length of stay in rehabilitation facilities has led to individuals with spinal cord injury (SCI) entering the community with unmet needs and fewer self-care skills to prevent secondary complications. The implementation of a self-management program for individuals with SCI for the management of these complex needs, including secondary complications, may be one option to fill these care gaps. A greater understanding of the meaning of self-management may facilitate the development of a tailored self-management program in this population. Thus, the current research aims to understand the meaning of self-management in traumatic SCI from the perspectives of individuals with traumatic SCI and their caregivers as well as acute care/trauma and rehabilitation managers.

**Methods:**

A descriptive qualitative approach was used. Semi-structured telephone interviews were conducted with 26 individuals with traumatic SCI, their family members/caregivers, and managers from acute care/trauma and rehabilitation centres. Inductive thematic analysis was applied.

**Results:**

The meaning of self-management in SCI related to two overarching themes of internal and external responsibility attribution and revealed differences between the meaning of self-management in SCI among individuals with traumatic SCI and their caregivers versus managers. Overall, the meaning of self-management among the SCI and caregiver participants related principally to internal responsibility attribution. For the manager participants, the meaning of self-management was much narrower and the overarching theme of internal responsibility attribution that was observed among the SCI-caregiver dyads was not as widely expressed by this group.

**Conclusions:**

Interventions that are co-created by users and health care professionals are associated with positive physical and mental health outcomes. Thus, the understanding of self-management from these varying perspectives could be applied to the development of a tailored self-management program that is relevant to individuals with traumatic SCI and their caregivers. This may involve the development of a program that uses some of the structure of traditional chronic disease self-management programs, in accordance with the beliefs held by the managers, but also incorporates elements of wellness/health promotion interventions, in accordance with the beliefs held by the SCI and caregiver participants.

## Background

The trend of decreasing length of stay in rehabilitation facilities has led to individuals with spinal cord injury (SCI) entering the community with unmet needs and fewer self-care skills to prevent secondary complications [1, 2]. Families and others comprising the informal support network for these people also have less time to adjust. These reduced lengths of stay in rehabilitation, and ensuing consequences, lead to higher rates of secondary complications and subsequent high rehospitalization rates [3–5]. Given this increasing emphasis on the community management of SCI, strategies that could be implemented in order to increase patients’ involvement and control of their medical treatment and its subsequent effects are required [6]. The implementation of a self-management program for individuals with SCI for the management of these complex needs, including secondary complications, may be one option to fill these care gaps, at least in part.

Self-management is commonly described as “…the individual’s ability to manage the symptoms, treatment, physical, and psychosocial consequences and lifestyle changes inherent in living with a chronic condition. Efficacious self-management encompasses the ability to monitor one’s condition and to affect the cognitive, behavioral, and emotional responses necessary to maintain a satisfactory quality of life” [7]. Self-management has been reported as enabling individuals to minimize pain, share in decision making about treatment, gain a sense of control over their lives [8, 9], reduce the frequency of visits to physicians, and enjoy a better quality of life [8, 10]. In SCI in particular, poor self-management has been identified as a significant factor in the development of an inactive lifestyle, secondary conditions, and de-conditioning [11, 12].

Hirsche and colleagues [13] conducted a qualitative study on the experiences of individuals with neurological conditions, including stroke, multiple sclerosis, as well as SCI, who participated in the Stanford Chronic Disease Self-Management Program (CDSMP). Participants with SCI reported the least satisfaction with the CDSMP. Individuals with SCI as well as some of the leaders of this self-management group suggested assembling a SCI-focused group (e.g., individuals with SCI needed information specific to and modules adopted for being in a wheelchair/reduced mobility). They also found that when attendant care is an important component (as is the case in individuals with SCI), a different approach may be needed to teach self-management skills (i.e., being a good director of care, instead of a person who manages care independently) [13]. More recently, Ide-Okochi and colleagues [14] examined the meaning of self-care and what factors influenced the construction of its interpretation among persons with cervical SCI living in Japan and determined that participants interpreted the meaning of self-care as being related to rehabilitation for independence in activities of daily living (ADLs); detachment from the body and self; embodiment; and, self-management. The theme of self-management included the sub-themes of internal locus of control, promoting health and well-being, and involving social interactions. The current research extends these previous studies i.e., [13, 14] and aims to understand the meaning of self-management in traumatic SCI from the perspectives of individuals with traumatic SCI and their caregivers as well as acute care/trauma and rehabilitation managers. This is the first study, to the best of our knowledge, to understand the meaning of self-management in traumatic SCI from such multiple perspectives, in a Canadian setting.

## Methods

### Design/approach

The present study took a descriptive qualitative approach using telephone interviews. This approach was employed as there is a paucity of research on self-management in individuals with traumatic SCI as well as their caregivers and the qualitative descriptive approach is well-accepted for researching topics about which little is known and yielding practical answers of relevance to policy makers and health care practitioners [15, 16]. Given the potentially important role that caregivers have in the self-management of individuals with SCI, as outlined above, individuals with traumatic SCI and their caregivers (“the SCI-caregiver dyad”) were included. Health care (or clinical) managers from adult acute care/trauma and rehabilitation centres were included in order to triangulate the findings from a health care professional and/or health system perspective (i.e., presumably, the managers would have both clinical and health system knowledge related to individuals with SCI and their families). Given the geographic diversity as well as the potential accessibility limitations of the study participants, telephone interviews were conducted. Using this approach, it is assumed that the current findings could be used to develop a tailored self-management program for individuals with traumatic SCI. Research ethics approval was obtained from the University of Toronto (Protocol Reference #26429). All participants provided informed consent prior to the interview.

### Recruitment

Community-based (i.e., non-hospital based) individuals with traumatic SCI were recruited via 1) an online advertisement posted on the SCI Canada-Ontario web site; 2) a print advertisement included in the SCI Canada-Ontario magazine “Outspoken”; 3) postings and direct personal interactions with Regional Services Coordinators from various SCI Canada-Ontario branches; and, 4) a community exercise rehabilitation program at McMaster University in Hamilton, Ontario (“MacWheelers”). Purposive sampling was used to identify and subsequently recruit study participants [17]. Some of the criteria for purposeful sampling included participants’ urban and rural status. Individuals with traumatic SCI who were interested in the study contacted the principal investigator by telephone or email to inquire about the study. Eligible participants included individuals who were 1) 18 years of age or older; 2) at least 12 months since injury; 3) fluent in English; 4) had experienced a traumatic SCI (e.g., a fall, motor vehicle accident, sporting accident, etc.); and, 5) who had a formal or informal caregiver who was willing to participate. It should be noted that the selection of a minimum of 12 months since injury was based on the findings of Hirsche and colleagues [13] who found that participation in the Stanford CDSMP less than one year after SCI may not be appropriate (i.e., readiness for information). Caregivers/family members were recruited via the individuals with traumatic SCI and were identified as the individual’s primary caregiver. Individuals with traumatic SCI and their family member/caregiver were interviewed separately to mitigate potential power imbalances, which would influence the experiences they would be willing to share. The contact information of managers from acute care/trauma and rehabilitation centres across Ontario that are recognized for treating individuals with SCI was identified via Internet searches. Managers were subsequently contacted by telephone, informed of the study, and asked whether or not they wished to be interviewed. Participants were recruited between September 2011 and May 2012. Recruitment ceased as the study approached the point of data saturation, which is the point when successive interviews become repetitive and no new responses or themes emerged [18].

### Data collection

Each participant took part in a semi-structured interview lasting approximately 60–75 min. The interviews were conducted by the principal investigator (SM). The interview guide consisted of semi-structured open-ended questions (see Table 1) and was pilot tested with a scientist experienced in qualitative methods (FW) as well as an individual with a SCI. Probes or recursive questioning were used during interviews to explore issues in greater depth and verify the interviewer’s understanding of the information being collected [18]. Examples of the open-ended questions from the interview guide are shown in Table 1. All interviews were digitally recorded and transcribed verbatim for data analysis.

**Table 1:** Interview guide for meaning of self-management in individuals traumatic spinal cord injury, their family members/caregivers, and acute care/trauma and rehabilitation managers (example: individuals with traumatic SCI guide)

Examples of open-ended questions from interview guide
1. Walk me through what you are currently doing to manage your condition?
2. How do you know you’re doing ok; that you can carry on your daily activities; are you satisfied with how you’re performing your daily activities?
3. What is self-management from your perspective (what comes to mind when you hear the phrase self-management)? *Probe: What is the ultimate goal of self-management?*
4. What are you currently doing to prevent any secondary complications, that is, any medical conditions that arise as a result of your spinal cord injury, such as urinary tract infections or pressure ulcers?

Example of probes: How so? Tell me more about that

### Data analysis

To facilitate the organization and analysis of the qualitative data, reflective notes from the interviews, as well as the transcripts were entered into NVivo 9 [19]. Analysis was conducted using inductive thematic analysis as described by Braun and Clark [20] to understand the meaning of self-management in traumatic SCI. Following verification of the accuracy of the transcripts by the interviewer, two researchers (FW, SJ) other than the principal investigator read a sample of the transcripts to become familiar with the data. The interview transcripts were initially coded manually by the principal investigator, giving full attention to all data. Following this, the codes were clustered into groups that shared similar meanings. At this point, three of the researchers (SM, FW, SJ) met to discuss the coding of a sample of the transcripts as well as the data assigned to the codes and themes/sub-themes. New themes and sub-themes were also discussed. Together, the researchers explored various thematic maps until consensus was reached.

## Results

### Description of participants

A total of 26 interviews were conducted, which included 7 individuals with traumatic SCI and 7 family/caregivers (i.e., 7 SCI-caregiver dyads), and 12 acute care/rehabilitation managers from across the province. Characteristics of the individuals with traumatic SCI are reported in Table 2. In terms of the family member/caregiver group, five were spouses (female), one was a sibling (male), and one was a personal support worker (female). The age range of the family members/caregivers was 39 to 65 years of age. All of the acute care/trauma and rehabilitation managers were female with an age range of 36 to 62 years of age and were either nurses or physical therapists. Overall, 7 of the 26 participants lived in Northern Ontario. To protect anonymity, quotes exemplifying the various themes only include the participant’s group (i.e., individuals with traumatic SCI, family member/caregiver, manager) and his or her sex.

**Table 2:** Characteristics of individuals with traumatic spinal cord injury

Characteristic	*N* = 7;
	n, Range
Sex	
Male	6
Female	1
Age	39–68
Time since injury (years)	2–25
Level of injury	
Paraplegia	5
Quadriplegia	2
Education	
<High school	2
Undergraduate/college	4
Post-graduate	1
Employment status	
Unemployed/retired	5
Part-time	1
Full-time	1

### Overview of themes

**Fig. 1:**
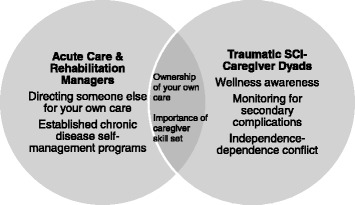
Meaning of self-management in traumatic spinal cord injury according to individuals with traumatic spinal cord injury, their spousal caregivers, and acute care/trauma and rehabilitation managers

In the current study, the meaning of self-management related to two overarching themes of internal responsibility attribution and external responsibility attribution (see Table 3). Responsibility attribution implies underlying and unexplored assumptions about who has responsibility and who assumes responsibility for self-management and health status. Responsibility is defined as taking on obligations to act in order to attain desired outcomes [21]. It is suggested that responsibility for disease management is attributed to different sources: some individuals assume responsibility (internal responsibility attribution) while others refer it to third parties such as employers, health care providers, or family members (external responsibility attribution) [22, 23]. Specifically, the sub-themes of wellness awareness, monitoring for secondary complications, independence-dependence conflict, directing someone else to provide your care, and ownership of your own care/empowerment in managing your own care comprised internal responsibility attribution. The sub-themes of established chronic disease self-management programs and the importance of caregiver skill set comprised external responsibility attribution. Furthermore, a clear delineation in the meaning of self-management was noted in the traumatic SCI and caregiver participants (i.e., the SCI-caregiver dyad) versus the manager participants (Fig. 1).

**Table 3:** Themes and sub-themes on the meaning of self-management in individuals with traumatic spinal cord injury according to individuals with traumatic spinal cord injury and their spousal caregivers and acute care/trauma and rehabilitation managers

Theme	Sub-themes
Internal responsibility attribution	Wellness awareness
	Monitoring for secondary complications
	Independence-dependence conflict
	Directing someone else to provide your care
	Ownership of your own care/empowerment in managing your own care
External responsibility attribution	Established chronic disease self-management programs
	Importance of caregiver skill set

### Internal responsibility attribution

#### Wellness awareness

Wellness awareness included lifestyle practices/changes including good nutrition, vitamin supplementation, exercise, and relaxation that these participants associated with living well and maintaining/optimizing health. This sub-theme is encapsulated by the following quotes:

*“Physical fitness, healthy eating, paying attention to what’s going on with the skincare, keeping your brain active, keep everything going, don’t spend too much time in front of the TV”* (SCI 6; Male with traumatic SCI).

*“Both of us have decided the best way is to exercise. So we’re exercising more, going on longer walks, trying to take walks instead of drive in cars short distances and that kind of thing, try to eat more healthy. I became a vegetarian about a year and a half and he’s about 85 %, 90 % vegetarian. We’re just more health-wise, that kind of thing”* (Caregiver 4; Wife of individual with traumatic SCI).

#### Monitoring for secondary complications

Participants described monitoring for secondary complications or being proactive about preventing secondary complications as a component of self-management in SCI. This monitoring or proactive behavior was often associated with a specific routine:

*“Then routines to my day, the washing up rituals. That used to be much quicker…Now it’s that I have to be dressed to protect the skin on my backside. I have to do that kind of ritual stuff in bed and that takes me roughly 20 min every morning. It bothers me but I know I need to do it so that there’s no skin breakdown or un-cleanliness or something like that doesn’t cause an issue down the road”* (SCI 4; Male with traumatic SCI).

Relatedly, many traumatic SCI participants underscored the importance of being aware of their bodies, with some participants describing the phenomenon of having to rediscover themselves post-injury:

*“…when you get a spinal cord injury you are now two people. There’s the upper part of you and there’s the lower part of you and they don’t communicate with each other. The lower part of you is like a little baby. It’s like it will react but you don’t know what that means because it’s not communicating with you. Like I mean it’s not directly linked to you. You can’t feel it. So you have to interpret what those reactions mean. Just like if a little baby is crying, well why is he crying, what’s going on?”* (SCI 5; Male with traumatic SCI).

It appeared that caregiver involvement was instrumental to these monitoring activities, especially for skin care:

*“But he’ll also be like ‘hey can you look at something it feels a little different’ because he’s very aware by just feeling. He does have mirrors but sometimes just feeling his skin he’s like what’s going on here and then I’ll check it out”* (Caregiver 4; Wife of individual with traumatic SCI).

#### Independence-dependence conflict

The sub-theme of independence-dependence conflict as a component of self-management emerged chiefly among individuals with traumatic SCI and their caregivers. Participants described this as being related to the ongoing attempt for independence on the part of individuals with traumatic SCI:

*“So what his success is I think it’s just a willingness to live and then to be autonomous and independent and we supported him in all that, in all those aspects and helped him buy a car. Two years after his accident we helped him buy a condo. He wanted to become autonomous”* (Caregiver 5; Brother of individual with traumatic SCI).

However, it was noted in some instances that in striving for this independence, there was a simultaneous risk of injury: “*… two of the times he* [individual with traumatic SCI] *was very tired, working long hours and did two transfers and ended up in injuries”* (Caregiver 4; Wife of individual with traumatic SCI).

(SCI 7; Male with traumatic SCI).

#### Directing someone else to provide your care

The sub-theme of directing someone else to provide your care (i.e., often a spouse for the prevention of secondary complications) was mainly put forward by acute care/trauma and rehabilitation managers. This sub-theme is encapsulated by the following quote:

*“I think that the biggest thing for the spinal cord injury is that whole directing their care and teaching them that what a great skill that is and how important it is because they’re going to have attendants all the time and to understand that part”* (Manager 5; Female Rehabilitation Manager).

Managers often linked an individual’s level of injury to his or her self-management abilities/behaviors:

*“So I’m going to say for a quadriplegic who is a complete injury it would be directing their own care in that they know how to direct caregivers to provide their care”* (Manager 11; Female Rehabilitation Manager).

#### Ownership of your own care/empowerment in managing your own care

The sub-theme of ownership of your own care or empowerment in managing your own care emerged as a component of self-management and was shared by all participant groups:

*“Then who’s reconnecting with them in the community and whose obligation is that? That sounds harsh but who is responsible? Is the rehab center responsible for how long in that transition? We certainly do follow people and connect with people but if they don’t come back, I can’t. So who owns that? I mean ultimately it’s the patient who owns it I guess. You’ve got to introduce people to it. You’ve got to give them a chance. If they’re never introduced to it, how can they own it”* (Manager 7; Female Rehabilitation Manager).

*“I guess I would interpret self-management as taking control of my health and taking the responsibility and making sure that I’m being responsible in terms of dealing with my health, whether it’s making sure that I book my yearly appointments and go to see my doctors. Like I said being proactive if there are issues that do arise, that I’m dealing with it right away and seeking out specialists if that’s needed to assist with whatever treatments or medications or something that I may need for it”* (SCI 1; Female with traumatic SCI).

Several participants believed that the ability to “take control” was associated with the individual’s own intrinsic psychological resources and thus varied from person to person.

### External responsibility attribution

#### Established chronic disease self-management programs

Among acute care and rehabilitation managers, the meaning of self-management in SCI was linked with existing or traditional chronic disease self-management models or programs, such as the Stanford CDSMP:

*“But it’s more around the philosophy of like the Stanford model and ownership of the chronic disease model. It’s a self-management model. That is what it’s based on”* (Manager 7; Female Rehabilitation Manager).

#### Importance of caregiver skill set

Lastly, several participants identified the importance of the caregiver’s own skill set in providing a wide range of support to the individual with SCI (e.g., basic and instrumental activities of daily living and assisting in the prevention/monitoring and/or management of secondary complications). They also linked this to the steps in self-management in SCI. The caregivers’ skill set was also shared across all the participant groups:

*“I mean some people will never be able to self-catheterize. So we educate their partner in care as to how they can help to do that. So they need to be taught at the same time as the individual patient. They need to know the risks in particular with you know I’m thinking of bladder dystonia and pressure sores, transferring and all of that. I mean these people aren’t going home to live by themselves. That’s quite rare. So they need to have the support service from their partner in care and family members and they need as much education as the patient does, sometimes more”* (Manager 4; Female Rehabilitation Manager).

*“I would have to do his catheter stuff and his bowel routine and all of that. I really wanted to be aware so that if issues came up with nursing, I knew what was involved you know…There have been times where you know even just from having a full bladder you don’t realize like if you’re not trained that you can look for signs”* (Caregiver 2; Wife of individual with traumatic SCI).

## Discussion

This study aimed to understand the meaning of self-management in traumatic SCI from the perspectives of individuals with traumatic SCI and their (mainly) spousal caregivers as well as acute care/trauma and rehabilitation health care (or clinical) managers. The meaning of self-management in SCI related to the two overarching themes of internal responsibility attribution and external responsibility attribution. Furthermore, a clear delineation in the meaning of self-management was noted in the traumatic SCI and caregiver participants (i.e., the SCI-caregiver dyad) versus the manager participants.

There is a paucity of research on responsibility related to disease management and where it does exist, it has been narrow in focus: rehabilitation after a hip fracture [23] and management of musculoskeletal pain [22]. Assuming responsibility is a key factor in the first stage of patient activation; the individual has to take responsibility before he/she can play an active part in managing disease [24]. Nevertheless, responsibility attribution among people with chronic illness has not been explored in detail and its influence on self-management has been rarely explored [21].

### Meaning of self-management in traumatic spinal cord injury and caregiver participants

For individuals with traumatic SCI and their caregivers, the meaning of self-management in SCI was largely reflected their belief in internal responsibility attribution. The sub-theme of ownership of one’s own care/empowerment in care management was central to the understanding of proper self-management by the traumatic SCI and caregiver participants. It was also described by manager participants, but not to the same extent as it was in the SCI-caregiver dyads. It is argued that the other sub-themes of wellness awareness, monitoring for secondary complications, and independence-dependence conflict also reflect internal responsibility attribution as some of these sub-themes correspond with the findings on internal responsibility attribution in a recent qualitative study [21]. For example, Audulv and colleagues [21] determined that those individuals who attributed responsibility to internal factors (e.g., beliefs and attitudes that one is an active agent in his or her own life) had a multi-faceted self-management regimen including a wide range of self-management behaviors in order to facilitate physical and mental well-being. It was further determined among those individuals who had a multi-faceted self-management regimen that there was an alternating between reflexive and routine strategies. With a reflexive strategy, self-management is closely evaluated and new information is sought and incorporated with an individual’s own experiences. With a routine strategy, self-management becomes a course of daily habits and routines. Thus, the themes identified by Audulv and colleagues [21] as being associated with internal responsibility attribution correspond with the sub-themes identified in the current study including monitoring for secondary complications (i.e., multi-faceted self-management regimen), which also involved specific routines (i.e., routine strategies) and a rediscovery of themselves post-injury (i.e., reflexive strategies), as well as wellness awareness (i.e., multi-faceted self-management regimen in order to facilitate physical and mental well-being). Wellness awareness as a component of the meaning of self-management according to the SCI and caregiver participants will be further discussed below as it contrasts to the manager participants’ meaning of self-management comprising established chronic disease self-management programs.

The sub-theme of independence-dependence conflict (including striving for independence) emerged as a component of the meaning of self-management and was consistent with the overarching theme of internal responsibility attribution among the traumatic SCI and caregiver participants. This sub-theme of independence-dependence conflict also comprised the notion that in striving for independence, individuals with traumatic SCI risked further injury or had experienced additional injuries. Indeed, the theme of independence (specifically, regaining independence in ADLs) also emerged in the study by Ide-Okochi and colleagues [14] on the meaning of self-care in persons with cervical SCI in Japan. Indeed, maintaining independence has been identified as a key component in the definition of self-management and healthy aging in other studies on individuals with neurological conditions (e.g., multiple sclerosis, stroke) [25, 26].

Finally, the sub-theme of the importance of caregiver skill set was observed in both the SCI-caregiver dyads as well as the manager participants, and was the one sub-theme among the SCI-caregiver dyads that related to external responsibility attribution.

### Meaning of self-management in acute care/trauma and rehabilitation managers

For the manager participants, the meaning of self-management was narrower than that perceived by the SCI/caregiver dyads and the overarching theme of internal responsibility attribution that was observed among the SCI-caregiver dyads was not as dominant in this group. The main sub-themes identified among the manager participants related to both internal and external responsibility attribution, which may reflect their belief in combined responsibility attribution in self-management. The sub-theme of directing someone else to provide your care was central to self-management in the manager participants. This theme relates to internal responsibility attribution as individuals with traumatic SCI were directing their own care and thus active agents in their own care and lives (i.e., rather than allowing others to determine their care). A few of the SCI-caregiver participants also related self-management to directing someone else to provide your care, consistent with the overarching theme of internal responsibility attribution observed in this group. In contrast to the current findings, Ide-Okochi and colleagues [14], identified the sub-theme of intended obedience, whereby SCI participants described family members as the ones who made decisions about daily regimens such as taking medications (i.e., versus the individual with SCI directing someone else to provide his or her care and/or joint decision making between the individual with SCI and the caregiver). In discussing this sub-theme, the authors noted cultural variations between Japan and America. In Japan, the family members of individuals with disabilities are expected to make important decisions instead of the patients themselves, while in America, individuals with even severe disabilities are encouraged to live independently. While caregivers play a significant role in the self-management of individuals with SCI [27], Ide-Okochi and colleagues [14] concluded that intended obedience (and decision making on the part of the family member alone) was not a suitable role for the individual with SCI. The sub-theme of the importance of caregiver skill set also comprised the meaning of self-management and was identified by both the manager and SCI-caregiver participants. Thus, despite the fact that individuals with SCI were directing their caregivers for their own self-management, they were dependent on the caregivers’ skills for this self-management, the latter reflecting external responsibility attribution. Audulv and colleagues [21] similarly determined that participants who attributed responsibility to external factors cited other people as critical for attaining success in self-management.

Manager participants reported that the meaning of self-management in SCI related to established chronic disease self-management programs, with some of the managers referencing the CDSMP. In the study by Audulv and colleagues [21], conventional self-management regimens (e.g., symptom control and management) were related to external responsibility attribution. Indeed, although the CDSMP includes several health behavior topics, the primary focus is on the daily control and management of disease [28]. In contrast, wellness interventions focus on maximizing health and quality of life [29]. It is argued that the manager participants’ reference to established or traditional chronic disease self-management programs versus the SCI and caregiver participants’ reference to wellness awareness speaks to the managers’ conventional notion of self-management in a SCI population. Furthermore, wellness/health promotion interventions are resources that allow the individual to choose behaviors to enhance and sustain quality of life within the context of living with a chronic disabling condition. Conversely, interventions primarily oriented toward controlling disease, symptoms, and risk factors have the chronic illness/disease perspective in the foreground, minimizing the wellness perspective and the associated element of patient choice [30]. Thus, the managers’ understanding of self-management in SCI as being associated with traditional chronic disease self-management programs is consistent with an external responsibility attribution, while the SCI and caregivers participants’ definition of self-management as comprising wellness awareness and the associated patient choice is consistent with an internal responsibility attribution. It should also be noted that the sub-theme of promoting health and well-being (health maintenance) was similarly noted by Ide-Okochi and colleagues [14]. However, the specific mechanisms needed to promote health and well-being or health maintenance were not included in this study, while in the current study, participants included lifestyle practices/changes including, good nutrition, vitamin supplementation, exercise, and relaxation as part of the meaning of self-management.

Finally, responsibility attribution may be more of a continuum from external to internal, rather than these defined groups. Future research may involve a quantitative examination of potential covariates or predictors to explain these attributions in self-management (e.g., for the development of programs that could be tailored to individual needs). Changes in responsibility attribution over time, particularly among the individuals with traumatic SCI themselves, would also be worthy of further study [21].

### Limitations

The current study acknowledges some limitations. In terms of the recruitment procedure, it is likely that a selection bias operated in those participants who agreed to take part in the research – they may have been healthier than those individuals who chose not to participate. Additionally, all participants had to have a caregiver who was willing to participate. The majority of traumatic SCI participants in the current study were male, which is consistent with the epidemiology of population-based studies e.g., [31], with female caregivers. Future research should attempt to focus on the perspective of females with a traumatic SCI as well as the perspectives of male caregivers in order to increase the applicability of the study findings. Future research should also seek to explore the self-management options for those individuals in the earlier stages post-injury (i.e., less than 1 year post-injury) and/or confirm that a self-management program at this stage is an inappropriate goal, as suggested by Hirsche and colleagues [13]. Finally, it is also important to note that options other than self-management programs may be worth exploring to minimize the risk of secondary complications including informational support (e.g., SCI-U, SCI Canada), peer support (e.g., SCI Canada), and/or other training/treatment for specific secondary complications (e.g., coping effectiveness training) [32].

## Conclusion

Interventions that are co-created by users and health care professionals are associated with positive physical and mental health outcomes [33]. Thus, the understanding of self-management from these varying perspectives could be applied to the development of a tailored self-management program that is associated with outcomes that are relevant to individuals with traumatic SCI and their family members/caregivers. This may involve the development of a program that uses some of the structure of traditional chronic disease self-management programs, in accordance with the beliefs held by the managers of the current study, but also incorporates elements of wellness/health promotion interventions, in accordance with the beliefs held by the SCI and caregiver participants. Such a program will thereby increase the empowerment and overall quality of life of individuals with traumatic SCI and their caregivers.

## Abbreviations

ADLs: activities of daily living
CDSMP: Chronic Disease Self-Management Program
SCI: spinal cord injury
